# HIV-1 Vif protein sequence variations in South African people living with HIV and their influence on Vif-APOBEC3G interaction

**DOI:** 10.1007/s10096-023-04728-0

**Published:** 2023-12-11

**Authors:** Monray Edward Williams

**Affiliations:** Human Metabolomics, North-West University, Potchefstroom, South Africa

**Keywords:** Polymorphisms, Vif, Subtype C, Sequencing

## Abstract

**Purpose:**

Despite extensive research, HIV-1 remains a global epidemic with variations in pathogenesis across regions and subtypes. The Viral Infectivity Factor (Vif) protein, which neutralizes the host protein APOBEC3G, has been implicated in differences in clinical outcomes among people living with HIV (PLHIV). Most studies on Vif sequence diversity have focused on subtype B, leaving gaps in understanding Vif variations in HIV-1C regions like South Africa. This study aimed to identify and compare Vif sequence diversity in a cohort of 51 South African PLHIV and other HIV-1C prevalent regions.

**Methods:**

Sanger sequencing was used for Vif analysis in the cohort, and additional sequences were obtained from the Los Alamos database. Molecular modeling and docking techniques were employed to study the influence of subtype-specific variants on Vif-APOBEC3G binding affinity.

**Results:**

The findings showed distinct genetic variations between Vif sequences from India and Uganda, while South African sequences had wider distribution and closer relatedness to both. Specific amino acid substitutions in Vif were associated with geographic groups. Molecular modeling and docking analyses consistently identified specific residues (ARGR19, LYS26, TYR30, TYR44, and TRP79) as primary contributors to intermolecular contacts between Vif and APOBEC3G, essential for their interaction. The Indian Vif variant exhibited the highest predicted binding affinity to APOBEC3G among the studied groups.

**Conclusions:**

These results provide insights into Vif sequence diversity in HIV-1C prevalent regions and shed light on differential pathogenesis observed in different geographical areas. The identified Vif amino acid residues warrant further investigation for their diagnostic, prognostic, and therapeutic potential.

**Supplementary Information:**

The online version contains supplementary material available at 10.1007/s10096-023-04728-0.

## Introduction

HIV-1 continues to be a global epidemic, affecting a significant number of individuals worldwide, with approximately 38.4 million people living with HIV (PLHIV) to date [1]. HIV-1 is classified into four major groups known as groups M, N, O, and P [2, 3]. Among these, group M is the most widespread and extensively studied. Group M can be further categorized into several subtypes, including A, B, C, D, F, G, H, J, and K [2, 3]. Each subtype displays distinct genetic characteristics and is often linked to specific geographical regions. Moreover, recombination events between different subtypes can lead to the emergence of circulating recombinant forms (CRFs) and unique recombinant forms (URFs) with diverse genetic compositions. Between 1990 and 2015, subtype C (HIV-1C) accounted for the highest global HIV prevalence, comprising 46.6% of cases. It was predominantly found in Southern Africa, Ethiopia, and South Asia (India) [4]. Subtype B (HIV-1B) had the second highest prevalence with 12.1% and was primarily associated with Western and Central Europe, North America, the Caribbean, Latin America, and Oceania. Lower HIV-1 incidence rates were observed for Subtype A (10.3%), CRF02_AG (7.7%), CRF01_AE (5.3%), subtype G (4.6%), and subtype D (2.7%) [4]. Other circulating recombinant forms (CRFs) accounted for 3.7% of cases, while unique recombinant forms (URFs) constituted 6.1% of cases. Combined, subtypes F, H, J, and K accounted for 0.9% of cases [4]. HIV-1 subtype variation can also be seen by subtype-specific changes in amino acid residues of the HIV-1 viral proteins.

The HIV-1 viral proteins, such as glycoprotein (gp)120, Transactivation of transcription (Tat), Viral protein R (Vpr), and Viral infectivity factor (Vif), exhibit subtype-specific variations, with certain amino acids being more prevalent in specific subtypes. Of particular interest in this study is the HIV regulatory protein Vif due to its importance in HIV-1 replication by counteracting the host cellular antiviral protein known as APOBEC3G (apolipoprotein B mRNA-editing enzyme catalytic polypeptide-like 3G) [5]. Vif is a relatively small cytoplasmic protein with a molecular weight of around 23 kDa and is composed of approximately 192 to 216 amino acids, depending on the HIV-1 subtype. The Vif protein consists of several functional domains and motifs. The tryptophan-rich stretch (amino acids 1–35) is the region that contains a high concentration of tryptophan residues and is essential for recognizing and interacting with the host protein APOBEC3G. The Vif APOBEC3F/G interaction domain (amino acids 42 and 43) is conserved and enables Vif to interact with APOBEC3F and APOBEC3G proteins. The RLRR nuclear localization inhibitory signal (amino acids 90–93) helps prevent the nuclear localization of Vif. The phosphorylation sites (S95, T96, S165, T170V, and T188) contain amino acids that can undergo phosphorylation, which may regulate Vif's function and interactions with other proteins. The Zinc-binding motif HCCH (H108, C114, C133, and H139) is involved in binding zinc ions, which is important for the structural stability and function of Vif. The SLQYLA motif (amino acids 144–149) plays a role in the antiviral activity of APOBEC3F and APOBEC3G against HIV-1. The PPLP Vif dimerization domain (amino acids 161–164) is responsible for promoting the dimerization of Vif protein [6]. These various domains and motifs within the Vif protein contribute to its functionality and interaction with host proteins and cellular processes.

APOBEC3G is cellular protein that has the ability to restrict HIV replication by causing hypermutation in the viral genome [7]. However, Vif counteracts this antiviral activity by binding to APOBEC3G and targeting it for degradation through the host cellular proteasome pathway [8]. By neutralizing APOBEC3G, Vif ensures the production of non-mutated, replication-competent viral particles. Therefore, functional Vif is crucial for HIV-1 replication, and the absence of this protein results in a significant impairment of HIV replication, leading to reduced viral infectivity [9]. Several studies have now shown that subtype-specific mutations in the Vif protein can alter the interaction with APOBEC3G, affecting its ability to degrade or neutralize the host antiviral factor.

A study has shown that the motif at residues 90–93 (RKKR) is thought to code for a nuclear localization inhibitory signal (NLIS) in subtype B (HIV-1B). However, in HIV-1C participants, the motif at this position is RLRR [10]. The arginine-rich motif is capable of nucleic acid interaction; however, the presence of Leucine at this position alters the positive charge and solubility of the protein at that site, thereby abrogating nucleic acid interactions [10]. In a HIV-1 long-term nonprogress or (LTNP), a mutant motif KKRK was identified, which demonstrated a typical nuclear localization signal (NLS) and facilitated predominant nuclear localization of Vif [10]. However, it is crucial for Vif to remain in the cytoplasm to perform functions such as APOBEC3G inactivation, as aberrant localization to the nucleus has been shown to significantly reduce viral infectivity [10]. Another study discovered a naturally occurring Vif mutant (I107T) that attenuates anti-APOBEC3G activity and HIV-1 replication [11]. In a study conducted in South Africa, it was found that all HIV-1C isolates contained a lysine (K) at the putative second phosphorylation site (T155). While the T155 phosphorylation site is not highly conserved [12] the substitution of T with K could potentially affect the phosphorylation and subsequently the activity of Vif [13]. Collectively, this highlights the fact that amino acid diversity of the Vif protein can influence its biological activity.

A major caveat of this line of research is that most studies examining these naturally occurring Vif mutations have been conducted in HIV-1B, and there is limited research on Vif protein sequence diversity in Southern Africa, where HIV-1C is predominant. The existing studies in South Africa have been conducted on small cohorts and have not explored the potential biological implications of these mutations [6, 13, 14]. Given that HIV-1C is responsible for the highest HIV-1 prevalence, it is crucial to understand how HIV-1C specific amino acid variations may influence HIV-1 pathogenesis. Furthermore, it is relevant to determine the biological role of these naturally occurring mutations in Vif-APOBEC3G binding.

Therefore, in this study, we aimed to (1) identify the natural occurring Vif protein sequence diversity in South African HIV-1C PLHIV, (2) to compare the observed sequence diversity in South Africa with other geographical regions where subtype C is prevalent, including Southern Africa, India, and Uganda, and (3) to employ molecular modelling techniques to investigate whether these subtype-specific variants have any impact on Vif-APOBEC3G interaction. Findings from this study may highlight key variants that may serve as diagnostic, prognostics and therapeutics targets as well as provide insight into the differential pathogenesis of HIV-1 across HIV-1C infection.

## Methods

### Study participants

The international Prospective Urban and Rural Epidemiology (PURE) study is a multinational prospective epidemiological study focused on assessing the development of cardiovascular diseases. In 2005 and 2010, baseline data collection took place in the North-West province of South Africa, with a sample size of 2010 and 1285 participants, respectively. HIV status was determined using the protocol of the South African Department of Health, which involved conducting the First Response rapid HIV card test (Premier Medical Corporation Limited, Daman, India). In cases where the test yielded a positive result, it was confirmed using the SD BIOLINE HIV 1/2 3.0 card test (Standard Diagnostics, INC, Korea). Once a positive result was confirmed, whole blood samples were sent to a health clinic for CD4^+^ cell count analysis using the flow cytometric method (Beckman COULTER, EPICS XlTM, Fullerton, USA) conducted by the National Health Laboratory Service. Informed consent was obtained for experimentation with human samples. The study protocol received approval from the Ethics Committee of the North-West University in Potchefstroom, South Africa (NWU-00106–22-A1).

For this specific study, a subset of *n* = 120 treatment-naïve participants was selected from the 2010 leg of the study. Out of this group, sequencing was successfully performed, and data was available for *n* = 51 participants (43%). These sequences, referred to as the HIV-1C SA study (HIV-1C SA-s), were included for further analysis. Additionally, we included 204 HIV-1C sequences from the Los Alamos database, which were obtained from countries where HIV-1C is prevalent. This includes 178 sequences from South Africa (HIV-1C SA-LA) in Southern Africa, 8 sequences from Uganda (HIV-1C UG-LA) in Eastern Africa, and 18 sequences from India (HIV-1C IND-LA) in South Asia.

### Laboratory assessment of blood

RNA was extracted from 200 μl of prepared plasma using the Quick-RNA™ Viral Kit (Zymo Research). Total RNA was reverse transcribed by reverse transcriptase-polymerase chain reaction (PCR) using the ProtoScript® II First Strand cDNA Synthesis Kit (New England Biolabs) and DNA was prepared for PCR analysis. The Tat exon 1/Vpr/Vif (HXB2 position 4900–6351) was amplified using the primer pair Vif-1 (5’GGGTTTATTACAGGGACAGCAGAG) / CATH- 4R (5’-GTACCCCATAATAGACTGTGACC), respectively. Amplification conditions were held at 94 °C for 2 min, followed by 40 cycles of denaturing (94 °C; 30 s), annealing (60 °C; 30 s), extension (68 °C; 2 min) and a final extension step for 10 min at 68 °C. Purification of all PCR products was performed with the Nucleospin® Gel and PCR clean-up kit, according to manufacturer instructions (Machery-Nagel GmbH & Co.KG, Germany). All PCR products were sequenced by BigDye Terminator v.3.1 Cycle Sequencing Ready Reaction Kit (ThermoFisher Scientific) and analysed on the ABI Prism 3130xl automated DNA sequencer (Applied Biosystems, Foster City, CA).

### Bioinformatic analysis of the clinical cohort: HIV-1C SA-s

Sequences were analysed using the Gene studio™ professional sequence analysis software (Version 2.2). Nucleotide sequences were translated into amino acid sequences using Expasy translate (Gasteiger et al. 2003). The study cohort was subtyped using COMET, which is an adaptive context-based modeling for ultrafast HIV-1 subtype identification [15].

### Bioinformatic analysis of database sequences: HIV-1C SA-LA, HIV-1C UG-LA and HIV-1C IND-LA

The HIV-1C Vif sequence datasets from South Africa, Uganda, and India were acquired from the Los Alamos database (https://www.hiv.lanl.gov/components/sequence/HIV/search/search.html) for the period spanning 2010 to 2023. The nucleotide sequences for each group was translated into amino acid sequences using Expasy translate (Gasteiger et al. 2003), and the corresponding amino acid sequences were identified for each sequence. Consensus amino acid sequence alignments were subsequently constructed for each respective group using Jalview.

### Phylogenetic analysis

Phylogenetic tree construction was conducted to examine the relationships among the clinical cohort (HIV-1C SA-s) and downloaded HIV sequences (HIV-1C SA-LA, HIV-1C UG-LA, and HIV-1C IND-LA). The analysis was performed using CLC Genomics Workbench v12.0 software (https://www.qiagen.com/). The software was used to import the sequences, and the "Create Alignment" tool with default settings (alignment mode = very accurate) was applied to generate a multiple sequence alignment.

To determine the most appropriate substitution model for constructing a maximum likelihood tree, the alignment underwent four different procedures supported by the "Model testing" tool: hierarchical likelihood ratio test (hLRT), Bayesian information criterion (BIC), Akaike Information Criterion (AIC), and Akaike Information Criterion corrected (AICc). These tests assessed various substitution models, including Jukes-Cantor (JC), Felsenstein 81 (F81), Kimura 80 (K80), Hasegawa-Kishino-Yano (HKY), and General Time Reversible (GTR).

Using the optimal substitution model identified during the model testing step (GTR with gamma rate variation and topology variation), the resulting alignment was employed to construct a maximum likelihood tree. The "Maximum Likelihood Phylogeny" tool was utilized, and the tree was built with 100-round bootstrap conditions. The tree was visualized in "cladogram" mode, grouping sequences based on their relatedness. Phylogenetic trees were constructed for Vif representative variants, including 4 HIV-1C SA-s, 3 HIV-1C SA-LA, 7 HIV-1C UG-LA, and 7 HIV-1C IND-LA. These representative variants were selected based on the criteria that the tree represents Southern and Eastern Africa as well as South Asia, regions where HIV-1C is predominant.

### Retrieval of HIV-1 Vif and the human APOBEC3G sequences

Consensus amino acid sequences were generated for each group, namely HIV-1C SA-s, HIV-1C SA-LA, HIV-1C UG-LA, and HIV-1C IND-LA, and these sequences were employed for downstream *in-silico* analysis (“Bioinformatic analysis of database sequences: HIV-1C SA-LA, HIV-1C UG-LA and HIV-1C IND-LA” section). The target sequence for this analysis was the Human APOBEC3G sequence, specifically residues 1–384, as specified by the Uniprot entry Q9HC16 (available at www.uniprot.org) (Supplementary Table 1). We acknowledge that Vif interacts with multiple binding partners; however, our study specifically focused on its major interacting partner, APOBEC3G, to elucidate the mechanisms underlying the variations in binding among Vif variants.

### Secondary structure prediction

The PSIPRED secondary structure prediction server (http://bioinf.cs.ucl.ac.uk/psipred) was utilized to predict the secondary structure elements, including alpha-helices, beta-sheets, and random coils, for HIV Vif variants and APOBEC3G [16]. Secondary structure prediction plays a crucial role as the initial step towards tertiary structure prediction. Additionally, it provides valuable insights into protein activity, relationships, and functions [17].

### Prediction of disordered state of Vif variants and APOBEC3G

The DISOPRED3 program (available at http://bioinf.cs.ucl.ac.uk/disopred [18]) was employed for protein disorder prediction and protein-binding site annotation within disordered regions. By submitting protein sequences, the server provides a probability estimate for each residue in the sequence indicating its likelihood of being disordered. In this study, the protein sequences of Vif variants and APOBEC3G were uploaded to the DISOPRED Prediction database for analysis.

### 3D structure prediction using I-TASSER

The interactive threading assembly refinement (I-TASSER) server is a comprehensive platform that employs a sequence-to-structure-to-function approach for automated protein structure and function prediction [19]. I-TASSER has been highly regarded and ranked as one of the top servers for generating accurate 3D protein structure predictions among various automated servers, as evidenced by its performance in the Critical Assessment of Structure Prediction (CASP10) [20]. The I-TASSER server utilizes multiple threading alignment to identify structural templates from the Protein Data Bank (PDB). These templates are then used for iterative structure assembly, resulting in the generation of predicted protein structures. Additionally, the predicted structure models are compared and matched with known proteins in function databases to derive functional insights [21]. This integrated process enables I-TASSER to provide valuable predictions for both protein structure and function.

In this study, the Vif and APOBEC3G protein sequences were submitted to the I-TASSER system, which is accessible at https://zhanggroup.org/I-TASSER/. During the prediction process, the C-score is used as an indicator of the likelihood of success for the predicted models. Higher C-score values indicate a higher level of confidence in the quality of the model [21]. The template modelling score (TM-score), on the other hand, is a sequence length-independent metric used to measure the structural similarity, ranging from zero to one [22]. A TM-score greater than 0.5 indicates a higher confidence level, while scores below 0.5 suggest lower confidence.

The following templates were used for the prediction of HIV-1C SA-s: 4n9f (sequence identity: 83% and coverage: 0.90, Normalized (Norm.) Z-Score: 10.09), HIV-1C SA-LA: 4n9f (sequence identity: 81% and coverage: 0.90, Norm. Z-score: 10.07), HIV-1C UG-LA: (sequence identity: % and coverage:, Norm. Z-score:), HIV-1C IND-LA: 4n9f (sequence identity: 81% and coverage: 0.90, Norm. Z-score: 10.14), APOBEC3G: 6p40A (sequence identity: 75% and coverage: 0.95, Norm. Z-score: 5.76). The templates above had the highest sequence identity, coverage and normalized Z score to the target sequences and were selected for model building.

### 3D structure quality assessment

To evaluate the quality of the predicted 3D structures, several structural parameters were assessed for each model. The Procheck and ERRAT tool from the Structural Analysis and Verification Server (SAVES) (https://saves.mbi.ucla.edu/) was utilized to determine if the predicted residues fell within the acceptable region of the Ramachandran plot [23]. Structures were deemed reliable if the majority (> 80%) of residues exhibited favourable distributions of phi and psi dihedral angles. ERRAT is a program for verifying protein structures determined by crystallography and the relative frequencies of noncovalent interactions between atoms of various types [24]. According to the generally accepted standard, a high-quality model is typically indicated by an ERRAT score greater than 50 [25, 26]. Furthermore, the root mean square deviation (RMSD) values were computed between the predicted structure and the homologous template structure using PYMOL/Maestro molecular visualization software. This step allowed for a comparison of the backbone structural similarity to the experimentally solved template structure. Structures with RMSD values below 2 Å were considered highly similar, indicating homology [27]. On the other hand, higher RMSD values indicated a lack of structural similarity between the predicted structures and templates. Protein structures that passed most, if not all, of the quality parameter tests were considered reliable for subsequent docking studies.

### Refinement and energy minimization

The predicted 3D structures were subsequently subjected to energy minimization using ModRefiner, an algorithm designed for high-resolution protein structure refinement [28]. The ModRefiner server refines the structure through a two-step process. In the first step, the central chain atoms are built using C alpha, followed by energy minimization. The second step involves building side chain rotamers while further refining the atoms [29]. The resulting model after energy minimization represents the conformation with the lowest energy minima for the protein structures, which are then utilized in molecular docking [29].

### Molecular docking: HADDOCK

The molecular docking of the Vif variants to APOBEC3G was performed using HADDOCK (High Ambiguity Driven protein–protein DOCKing) webserver (Version 2.2) in the EASY interface available at https://wenmr.science.uu.nl/haddock2.4/. The key regions for the interaction between APOBEC3G-Vif have been identified, which include 126-FWDPDYQ-132 of APOBEC3G and 22-KSLVK-26, 40-YRHHY-44, 69-YWGL-72 of Vif [30–34]. Therefore, these were given as input active site residues to specify the search space for the docking simulation. The docked structures were organized into clusters, and each cluster was assigned several parameters, including a HADDOCK score, cluster size, RMSD from the overall lowest energy conformations, Z-score, buried surface area, and bonding energies such as Vander Waal's, electrostatic, desolvation, and restraints violation energies. Among these clusters, we identified and selected the best-docked complex for further analysis. The selection was based on the topmost cluster suggested by HADDOCK, which exhibited the desired residual interactions. To assess the binding affinity (ΔG) and dissociation constant (Kd) of the docked complexes, the PRODIGY webserver was utilized. The PRODIGY webserver, available at https://wenmr.science.uu.nl/prodigy/, employs inter-molecular contacts within a distance cut-off of 5.5 Å to predict binding affinities [35]. Using this tool, the binding affinities and dissociation constants of the docked complexes were calculated.

### Statistical analysis

All analyses were conducted using SPSS (version 27, IBM, USA). The chi-squared test was utilized to compare groups, and a significance level of *p* < 0.005 was deemed as statistically significant.

### Accession numbers for Vif variants

The Vif sequences from South Africa reported in this study were deposited in GenBank under accession numbers OR194556-OR194606.

## Results

### Clinical data of HIV-infected participants

This sub-study included *n* = 51 PLHIV from South Africa who participated in the 2010 leg of the PURE study. These participants were recruited from the North-West province in South Africa. The average age of the participants was 48.196 (± 7.54), and the majority of them were female (*n* = 38, 75%). At the time of sample collection, all participants were treatment-naïve, and their mean CD4 count was 297.41 (± 147.3) cells/mm3.

### Phylogenetic analysis

**Fig. 1:**
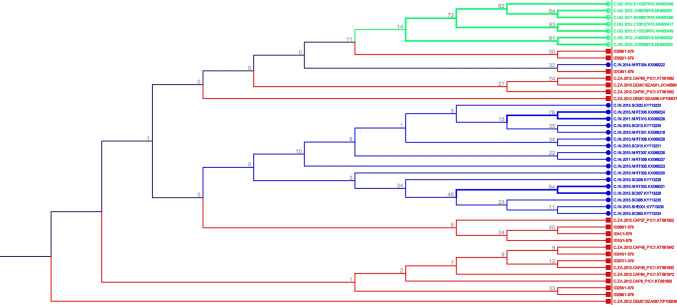
The maximum likelihood phylogenetic tree analysis representing the similarities on the Vif gene of HIV-1C from South Africa (blue), India (red) and Uganda (green). The bootstrap probability (> 60%, 100 replicates) are highlighted

Sanger sequencing was employed to analyse the Vif genetic variations in all HIV-1C SA-s participants. HIV-1 subtyping revealed that all participants from the clinical cohort HIV-1C SA-s of this study belonged to subtype C. To assess sequence similarity, the Vif sequences from the clinical cohort (HIV-1C SA-s) were compared to Vif sequences from the Los Alamos database representing South Africa (HIV-1C SA-LA), Uganda (HIV-1C UG-LA), and India (HIV-1C IN-LA). These sequences represented variations observed in Southern Africa, Eastern Africa, and South Asia, respectively. A phylogenetic tree was constructed using the maximum likelihood method, with representative variants included (Fig. 1). The Vif sequences from India (blue) and Uganda (green) appeared less closely related to each other, while the South African sequences (red) exhibited broader distribution and were found to be closely related to both Ugandan and Indian sequences (Fig. 1). The phylogenetic tree including all sequences can be found in the Supplementary Fig. 1.

### Vif protein sequence alignment

**Fig. 2:**
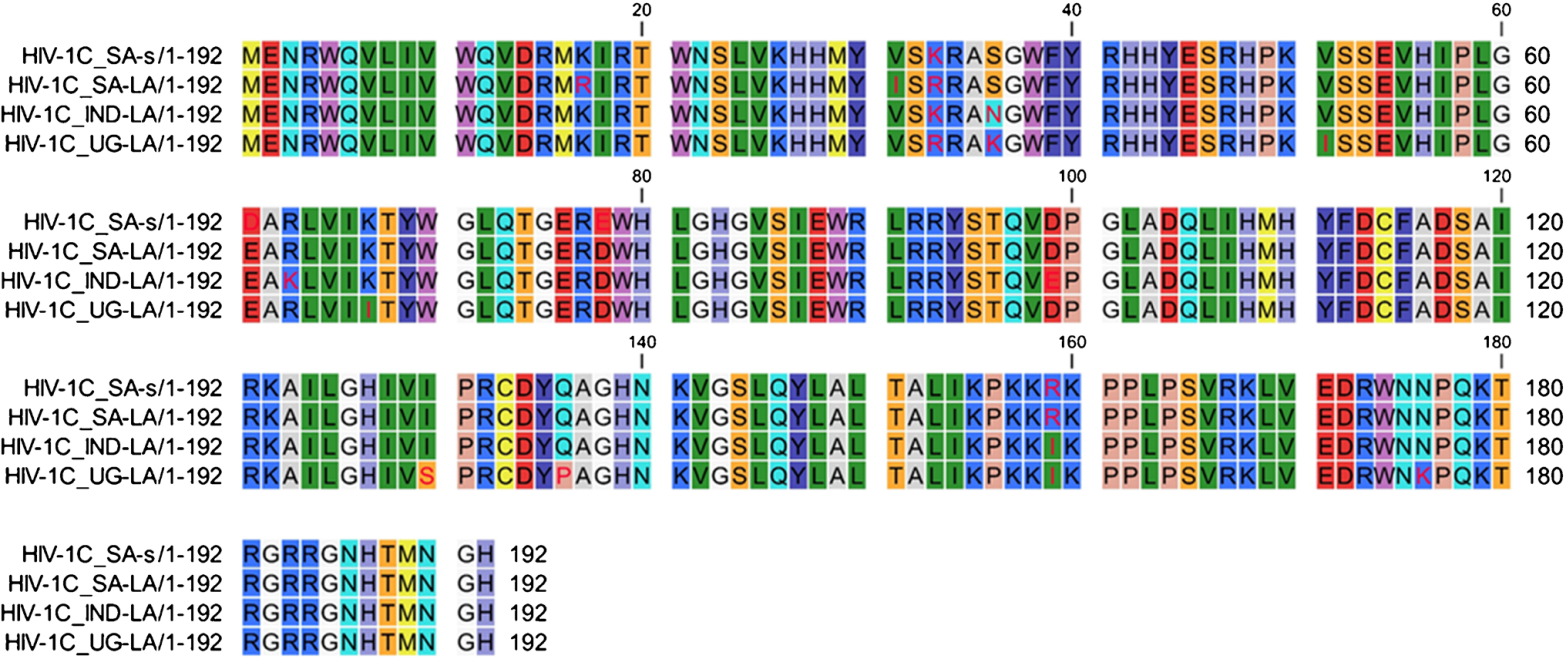
Multiple sequence alignment of consensus Vif protein sequences from HIV-1C SA-s, HIV-1C SA-LA, HIV-1C UG-LA and HIV-1C IN-LA. Sequence variation amin acids between groups were displayed in red

The nucleotide sequence was translated into amino acid sequence for the Vif protein, and the consensus sequence for each group was used to determine sequence variation (Fig. 2). From the consensus sequence alignment, several variants are highlighted which include K17R (HIV-1C SA-LA), V31I (HIV-1C SA-LA),V51I (HIV-1C UG-LA), E61D (HIV-1C SA-LA), R63K (HIV-1C IN-LA), D78E (HIV-1C SA-LA), D99E (HIV-1C IN-LA), I129S (HIV-1C UG-LA), Q135P (HIV-1C UG-LA), R159I (HIV-1C SA-s, HIV-1C SA-LA) and I159K (HIV-1C UG-LA and HIV-1C IN-LA) and N176K (HIV-1C UG-LA) (Fig. 2).

Furthermore, we aimed to determine the frequencies of specific variants in each group based on the sequences investigated, as shown in Table 1. The K17R substitution was found to be more common in South African participants and sequences, particularly in HIV-1C SA-s (45%) and HIV-1C SA-LA (87%), compared to Ugandan and Indian HIV-1C sequences (*p* < *0.001*). On the other hand, the V31I substitution was more prevalent in HIV-1C SA-LA sequences compared to the other groups (*p* < *0.001*). The D99E substitution was predominantly found in Indian HIV-1C sequences, with negligible occurrence in the other groups (*p* < *0.001*). The I130S substitution was highly prevalent in Ugandan HIV-1C sequences (86%), while being less common in Indian (44%) and South African sequences (20% and 29% respectively) (*p* = *0.002*). Additionally, the I159K and N176K substitutions were also more prevalent in the Ugandan sequences compared to the other groups (*p* < *0.001*) (Table 1).

**Table 1:** Frequency of HIV-1 Vif variants in HIV-1C

Vif residue variant	HIV-1C SA-s	HIV-1C SA-LA	HIV-1C UG-LA	HIV-1C IN-LA	*p*
	*N* = *51*	*N* = *138*	*N* = *7*	*N* = *18*	
K17R	23 (45%)	120 (87%)	3 (43%)	3 (17%)	** < .001**
V31I	13 (25%)	99 (72%)	I (14%)	3 (17%)	** < .001**
V51I	14 (27%)	59 (43%)	4 (57%)	8 (44%)	.019
E61D	26 (51%)	57 (41%)	6 (86%)	5 (28%)	.040
R63K	9 (18%)	26 (19%)	2 (29%)	9 (50%)	.019
D78E	27 (53%)	65 (47%)	2 (29%)	3 (17%)	.006
D99E	1 (2%)	2 (1%)	0 (0%)	16 (89%)	** < .001**
I130S	10 (20%)	40 (29%)	6 (86%)	8 (44%)	**.002**
Q136P	4 (8%)	34 (25%)	4 (57%)	3 (17%)	.007
R159I	13 (25%)	67 (49%)	4 (57%)	12 (67%)	.006
I159K	0 (0%)	1 (1%)	3 (43%)	1 (5%)	** < .001**
N176K	20 (39%)	23 (17%)	7 (100%)	5 (28%)	** < .001**

Significant findings are in bold

### Molecular modelling

**Fig. 3:**
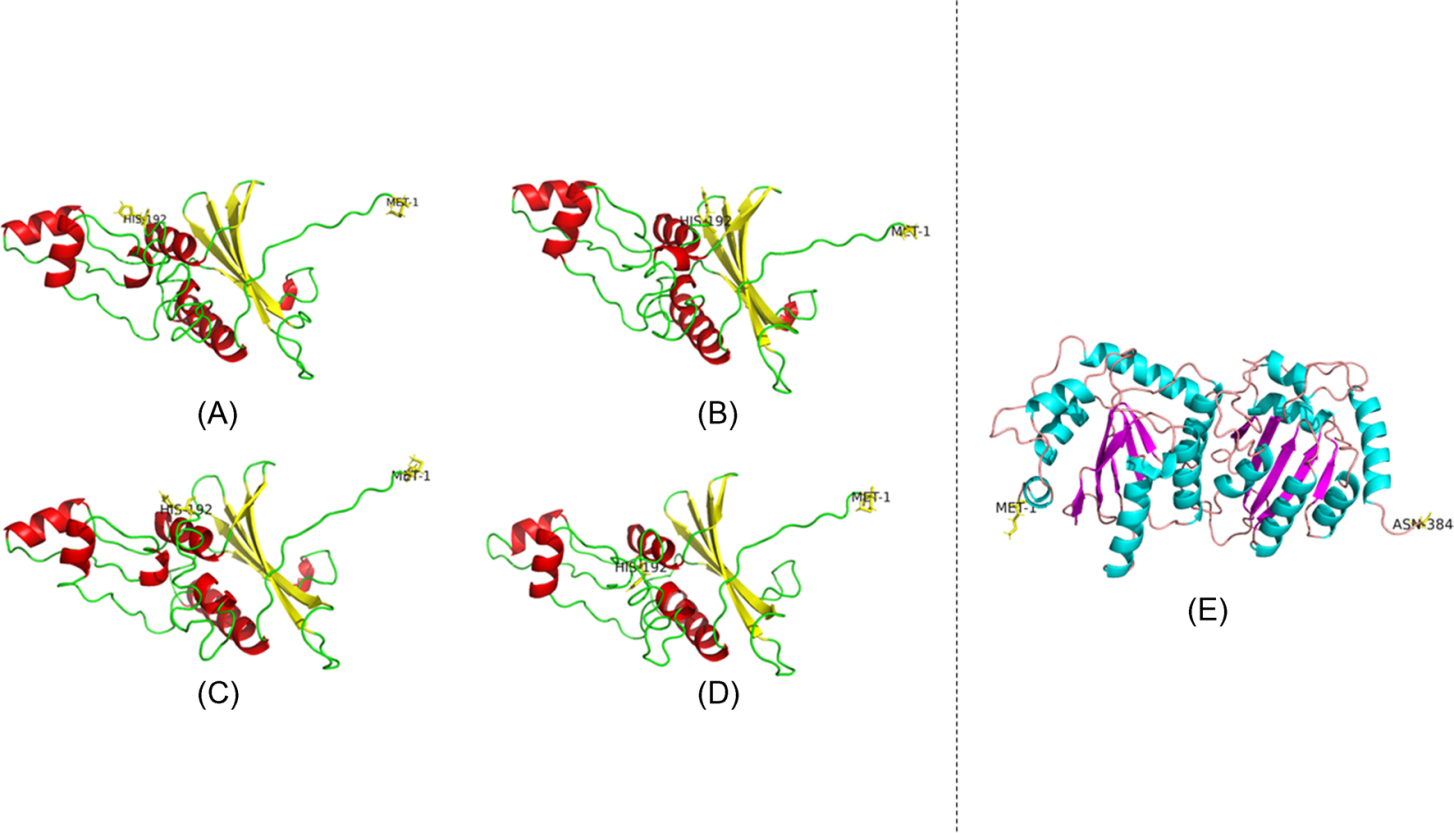
Model of The Vif proteins. **A** Vif HIV-1C SA-s, **B** HIV-1C SA-LA, **C** HIV-1C UG-LA and (**D**) HIV-1C IND-LA. The alpha helices are indicated in red and beta strands are indicated in yellow. The N-terminal Met-1 and C-terminal His-192 are indicated as yellow sticks

According to the secondary structure prediction, Vif HIV-1C SA-s was expected to have seven β-strands, seven α-helices, one disordered state, and one disordered state with protein binding (Supplementary Fig. 2A). Vif HIV-1C SA-LA was predicted to have seven β-strands, eight α-helices, one disordered state, and one disordered state with protein binding (Supplementary Fig. 2B). Vif HIV-1C_UG-LA was predicted to have six β-strands, seven α-helices, and one disordered state with protein binding (Supplementary Fig. 2C). Vif HIV-1C_IN-LA was predicted to have seven β-strands, seven α-helices, and one disordered state with protein binding (Supplementary Fig. 2D). However, based on the 3D predicted models, both Vif HIV-1C SA-s and Vif HIV-1C SA-LA exhibited five β-strands and seven α-helices. Vif HIV-1C UG-LA displayed five β-strands and seven α-helices (Fig. 3A-D), while Vif HIV-1C IN-LA showed five β-strands and five α-helices (Fig. 3A-D). Furthermore, APOBEC3G was predicted to have ten β-strands and sixteen α-helices (Supplementary Fig. 1E), but it was found to have eleven β-strands and fourteen α-helices (Fig. 3E).

Furthermore, all 3D predicted structures exhibited favourable C-scores within the upper range, indicating high confidence in the models. Additionally, all models achieved TM-scores greater than 0.5, indicating correct topology (Table 2). To ensure further quality assessment, we subjected the models to additional evaluations such as ERRAT and Procheck. All models obtained scores above 50 in ERRAT, indicating high-quality models. Moreover, all models passed Procheck, with more than 80% of residues falling within the allowed regions. Lastly, all Vif variants had RMSD scores less than 2 Å when compared to the homologous templates suggesting high structural similarity (Table 2). The APOBEC3G had a RMSD slightly higher than 2 Å (Table 2).

**Table 2:** Summary of the quality assessment scores for the 3D predicted structures of Vif variants and APOBEC3G

Protein name	Template	I-TASSER		ERRAT	Procheck residues (percentage in allowed region)	RMSD ( Å)
		Estimated TM-Score	C-score			
HIV-1C SA-s	4n9f	0.69 ± 0.12	−0.15	89.6175	97.6	0.695
HIV-1C SA-LA	4n9f	0.70 ± 0.12	−0.11	91.3043	98.2	0.642
HIV-1C UG-LA	4n9f	0.70 ± 0.12	−0.10	92.3077	98.2	0.643
HIV-1C IND-LA	4n9f	0.69 ± 0.12	−0.16	83.6957	98.2	0.633
APOBEC3G	6p40A	0.81 ± 0.09	0.71	93.617	98.6	3.449

### Molecular docking: HDDOCK

When comparing the binding of Vif variants to APOBEC3G, it was observed that Vif HIV-IN-LA exhibited the highest predicted binding affinity, with a docking score of -207.1 ± 1.9, a binding affinity of -14.6 kcal/mol, and a Kd of 2.1 × 10–11 M. Furthermore, Vif HIV-IN-LA demonstrated the highest number of intermolecular contacts (ICs) with APOBEC3G, totaling 98 ICs (Table 3). Following Vif HIV-IN-LA, the Vif variants HIV-1C SA-LA, HIV-1C UG-LA, and HIV-1C SA-s showed progressively decreasing binding affinities (Table 3).

**Table 3:** The predicted binding affinities and number of interactions between APOBEC3G and Vif variants

Vif variant	HADDOCK Score	ΔG kcal mol^−1^	K_d_ (M)	Number of intermolecular contacts (ICs)
HIV-1C SA-s	−156.9 ± 7.7	−10.2	3.4 × 10^–8^	Total: 71 charged-charged: 11 charged-polar: 4 charged-apolar: 29 polar-polar: 1 polar-apolar: 4 apolar-apolar: 22
HIV-1C SA-LA	−184.1 ± 7.2	−14.2	4 × 10^–11^	Total: 87 charged-charged: 13 charged-polar: 5 charged-apolar: 39 polar-polar: 1 polar-apolar: 16 apolar-apolar: 13
HIV-1C UG-LA	−170.1 ± 4.2	−11.1	6.9 × 10^–9^	Total: 79 charged-charged: 12 charged-polar: 4 charged-apolar: 31 polar-polar: 1 polar-apolar: 7 apolar-apolar: 24
HIV-1C IN-LA	−207.1 ± 1.9	−14.6	2.1 × 10^–11^	Total: 98 charged-charged: 17 charged-polar: 8 charged-apolar: 42 polar-polar: 0 polar-apolar: 14 apolar-apolar: 17

Number of ICs at the interface within the threshold distance of 5.5 Å

In the case of the interaction between HIV-1C IN-LA and APOBEC3G, a total of 98 ICs were reported. Among these ICs, the Vif amino acids ARG19 (9), LYS26 (6), HIS43 (6), TRP79 (5), and TRP174 (5) made the most significant contributions to the interaction with APOBEC3G (Supplementary Table 2). For HIV-1C SA-LA, the Vif amino acids LYS26 (6), TYR30 (8), PHE39 (6), HIS42 (8), TYR44 (9), and ARG47 (6) were identified as the major contributors to the ICs (Supplementary Table 3). In the case of HIV-1C UG-LA, the Vif amino acids ARG19 (7), TYR30 (5), TYR44 (10), and TRP70 (5) were found to be the primary contributors to the ICs (Supplementary Table 4). Lastly, for HIV-1C SA-s, the Vif amino acids MET16 (6), ARG19 (7), TYR44 (7), TRP79 (5), and LYS160 (6) were observed to have significant contributions to the ICs (Supplementary Table 5).

Consistently across the different Vif variants, the residues ARG19, LYS26, TYR30, TYR44, and TRP79 were identified as the main contributors to the majority of intermolecular contacts (IC) between Vif and APOBEC3G.

**Fig. 4:**
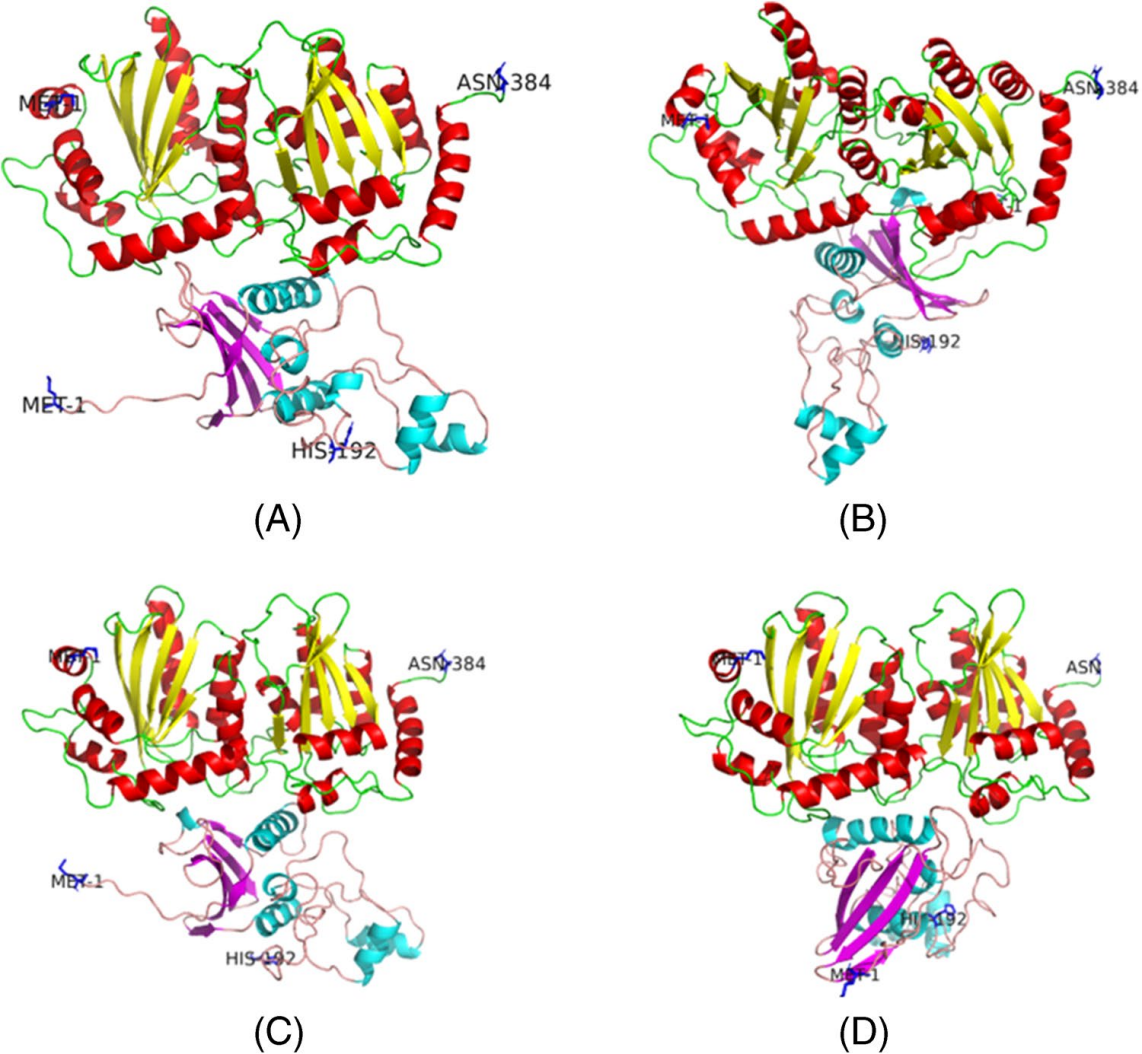
The top predicted binding poses for APOBEC3G docked to Vif variants. **A** HIV-1C SA-s, **B** HIV-1C SA-LA, **C** HIV-1C UG-LA and (**E**) HIV-1C IN-LA. N and C terminal amino acids are labelled in blue sticks with residue name

All Vif variants were found to bind to the same binding pocket of APOBEC3G, as illustrated in Fig. 4. The binding orientation of Vif HIV-1C SA-s (Fig. 4A) and HIV-1C UG-LA (Fig. 4C) to APOBEC3G was similar to each other. On the other hand, the orientation of HIV-SA-LA (Fig. 4B) and HIV-1C IN-LA (Fig. 4D) differed slightly in comparison to the other groups. The similarity in orientation between HIV-1C SA-s (Fig. 4A) and HIV-1C UG-LA may provide an explanation for the similar binding scores and affinities observed in these groups. In contrast, HIV-SA-LA and HIV-1C IN-LA Vif exhibited distinct binding orientations. Notably, HIV-1C IN-LA displayed a more tightly fitted binding to APOBEC3G, which could account for the greater number of intermolecular contacts (ICs), as well as the higher binding scores and affinities observed in this group.

## Discussion

This study has yielded three notable findings that warrant further discussion. Firstly, the phylogenetic analysis revealed distinct genetic variations between Vif sequences from India and Uganda, while South African sequences exhibited a broader distribution and demonstrated closer relatedness to both Ugandan and Indian sequences. This suggests that South African sequences may possess greater genetic diversity compared to Indian and Ugandan HIV-1C sequences. Secondly, specific amino acid substitutions in the Vif protein were significantly associated with particular geographic groups. Notably, K17R and V31I substitutions were more prevalent in South African sequences, while D99E was significantly more common in Indian sequences. In contrast, I130S, I159K, and N176K substitutions were found to be significantly more prevalent in Ugandan HIV-1C sequences. Thirdly, Vif HIV-1C IN-LA exhibited the highest predicted binding affinity to APOBEC3G among the groups studied, with a docking score of -207.1 ± 1.9, a binding affinity of -14.6 kcal/mol, and a Kd of 2.1 × 10–11 M. Among the different Vif variants, the residues ARGR19, LYS26, TYR30, TYR44, and TRP79 consistently emerged as the primary contributors to the majority of intermolecular contacts (ICs) between Vif and APOBEC3G. This finding underscores the significant functional role of these amino acids in the interaction between Vif and APOBEC3G.

To date, the majority of molecular analyses on HIV-1 Vif proteins have focused on subtype B-derived Vif. However, it is worth noting that Vif proteins derived from subtype C strains have demonstrated the highest activity against APOBEC3G compared to subtypes A, B, CRF01_AE, and CRF02_AG [36]. This was ascribed to its increased its binding activity to APOBEC3G [36]. Gaining a comprehensive understanding of how HIV-1C Vif variation influences interactions with APOBEC3G is crucial for elucidating the pathogenesis of HIV-1C.

Previous studies have characterized Vif from South Africa [6, 13, 14] and India [37] respectively. However, to the best of our knowledge, this is the first study to characterize Vif from different geographical regions where HIV-1C predominates. Interestingly, our findings reveal that despite all sequences investigated belonging to HIV-1C, the Vif gene sequences from India displayed distinct genetic variations compared to the sequences from Uganda. This emphasizes the significance of considering regional variations, beyond subtype differences alone, when investigating the impact of viral proteins on pathogenesis.

South African sequences showed a closer genetic relationship to both India and Uganda, whereas Indian and Ugandan sequences exhibited greater genetic differences from each other. These findings suggest that South African HIV-1C sequences may possess higher genetic diversity compared to those from India and Uganda, respectively. A previous study also observed a similar pattern, where HIV-1C from Southern African countries displayed genetic distinctiveness from Southeast Asian HIV-1C. Furthermore, the Southern African HIV-1C variants frequently displayed a dicysteine motif, which is a neurotoxic amino acid signature within the HIV protein known as Tat [38]. Consistent with these findings, studies have demonstrated that higher HIV-1 genetic diversity is correlated with more severe clinical outcomes, such as AIDS progression and neuropsychological impairment [39] well as reduced treatment effectiveness[40]. These observations may provide an explanation for the variations in clinical outcomes among PLHIV and the notably high prevalence of HIV-1 cases in Southern Africa.

On the protein level, certain amino acid variations were found to be more prevalent in HIV-1C sequences from specific geographic regions. Notably, the amino acid substitutions K17R and V31I were specifically associated with South African HIV-1C sequences. Additionally, a separate study reported that the amino acids K17 and V31 were not derived from subtype C Vif sequences, which aligns with the findings mentioned in this study [36]. In the same study, it was observed that the introduction of a K17R mutation resulted in a significant decrease in viral infectivity. This finding suggests that the amino acid R17 plays a critical role in binding to APOBEC3G [36]. Furthermore, the study revealed that the V31I mutation promotes cell cycle arrest by inducing the degradation of protein phosphatase 2 regulatory subunit B family (PPP2R5) subunits. This suggests that the V31I mutation has an impact on the regulation of cell cycle progression through the modulation of PPP2R5 subunit levels [41, 42]. In addition to its functional role in cell cycle arrest, we propose that the V31I mutation in HIV-1C sequences may also contribute to APOBEC3G binding. However, it is important to note that further investigation is needed to fully understand the impact and significance of the V31I mutation in APOBEC3G binding.

The Vif amino acid region from 85 to 99 and 169 to192 is also shown to mediate Vif-APOBEC3G binding [37, 43]. In this study, we present evidence of the high prevalence of the D99E mutation in Indian HIV-1C sequences. This finding aligns with a similar observation reported in a previous study [37] where the authors suggested a potential association between this mutation and viral infectivity [37]. According to the findings reported in our study, it is possible that D99E is one of several amino acid substitutions that contribute to an increased binding affinity to APOBEC3G, consequently influencing viral infectivity. I130S, I159K, and N176K substitutions were found to be significantly more prevalent in Ugandan HIV-1C sequences. Previous studies have indicated that the N-terminal amino acids of Vif play a critical role in binding to APOBEC3G. However, other research has suggested that the C-terminal amino acids of Vif might be involved in the interaction between Vif and the core binding factor (CBF)-β, as well as in the assembly of the Cullin (CUL)5-containing E3 ligase complex [44]. However, the exact functional roles of I130S, I159K, and N176K substitutions requires further investigation.

In our study, we employed molecular docking techniques to identify the key residues involved in the interaction between Vif and APOBEC3G. Through this analysis, we identified specific amino acids that contribute to the most intramolecular interactions between these proteins which included ARG19, LYS26, TYR30, TYR44, and TRP79. Our findings corroborate previous studies indicating that LYS26, TRY30, and TYR44 are located within the region of Vif that is crucial for suppressing the activity of APOBEC3G [31, 45–47]. Our study acknowledges that ARG19 may have importance in binding APOBEC3G. However, it is worth noting that previous research has shown that a mutation from arginine to lysine at position 19, which is present in subtype B, did not have a significant effect on viral infectivity [36]. The TRP79 residue in the Vif protein is highly conserved across various HIV-1 subtypes, indicating its potential functional significance. Intriguingly, studies have demonstrated that TRP79 plays a more critical role in the degradation of APOBEC3C by Vif [47, 48]. However, it is noteworthy that TRP79 is not as essential for Vif-mediated degradation of APOBEC3G or its exclusion from virions [47, 48].

Lastly, we found that Vif HIV-1C IN-LA had the greatest binding affinity to APOBEC3G in comparison to other geographical regions where HIV-1C predominates. Considering the higher prevalence virulence of HIV-1 in Southern Africa, it was expected that Southern African Vif to have the highest predicted binding affinity to APOBEC3G. This was shown in the case of other viral proteins including Tat and gp120, whereby these proteins had amino acid features that represented lower levers of virulence in India compared to Southern Africa [38, 49]. It is important to acknowledge that the number of Southern African sequences analyzed in this study was significantly higher compared to sequences from other geographical regions. It may also mean that due to the genetic diversity of Southern African sequences, it may possess the capacity to evade immune surveillance through various mechanisms beyond solely binding to APOBEC3G.

This observation underscores the critical need for further studies aimed at characterizing HIV-1 infections in regions where subtype C is prevalent. Such regions have been relatively understudied and exploring the unique genetic characteristics and viral-host interactions in these areas will significantly contribute to our understanding of the virus. Advancing research in this under-investigated field is crucial for the development of effective prevention and treatment strategies for HIV-1C infections.

## Conclusion

In this study, we conducted a characterization of Vif protein sequence variation in geographical regions where HIV-1C subtype C predominates, namely Southern Africa, Eastern Africa, and Southeast Asia. Our findings revealed a distinct genetic variation between Vif sequences from India and Uganda, while South African sequences displayed a wider distribution and demonstrated closer relatedness to both the Ugandan and Indian sequences. Specific amino acid substitutions in the Vif protein were found to be significantly associated with particular geographic groups. To gain further insights, we employed molecular modeling techniques to generate accurate 3D structures of the Vif proteins. Molecular docking of Vif variants to APOBEC3G indicated that among the different variants of Vif, the residues ARGR19, LYS26, TYR30, TYR44, and TRP79 consistently emerged as the main contributors to the majority of intermolecular contacts between Vif and APOBEC3G. Additionally, Vif HIV-1C IN-LA (India) exhibited the highest predicted binding affinity to APOBEC3G among the groups studied. The protein signatures highlighted in this study should be further investigated to fully characterize the function of HIV-1C Vif and establish its prognostic, diagnostic, and therapeutic potential.

### Supplementary Information

Below is the link to the electronic supplementary material.

Supplementary file1 (TIF 317 kb)

Supplementary file2 (DOCX 467 kb)

Supplementary file3 (DOCX 16 kb)

Supplementary file4 (DOCX 23 kb)

Supplementary file5 (DOCX 23 kb)

Supplementary file6 (DOCX 20 kb)

Supplementary file7 (DOCX 20 kb)

## Data Availability

All data used in this study is attached to this manuscript.
