# Supplementary figures and images for: HIV-1 Vif protein sequence variations in South African people living with HIV and their influence on Vif-APOBEC3G interaction
Source: Eur J Clin Microbiol Infect Dis. 2023 Dec 11;43(2):325–38. doi: 10.1007/s10096-023-04728-0 (PMC10821834; doi:10.1007/s10096-023-04728-0)

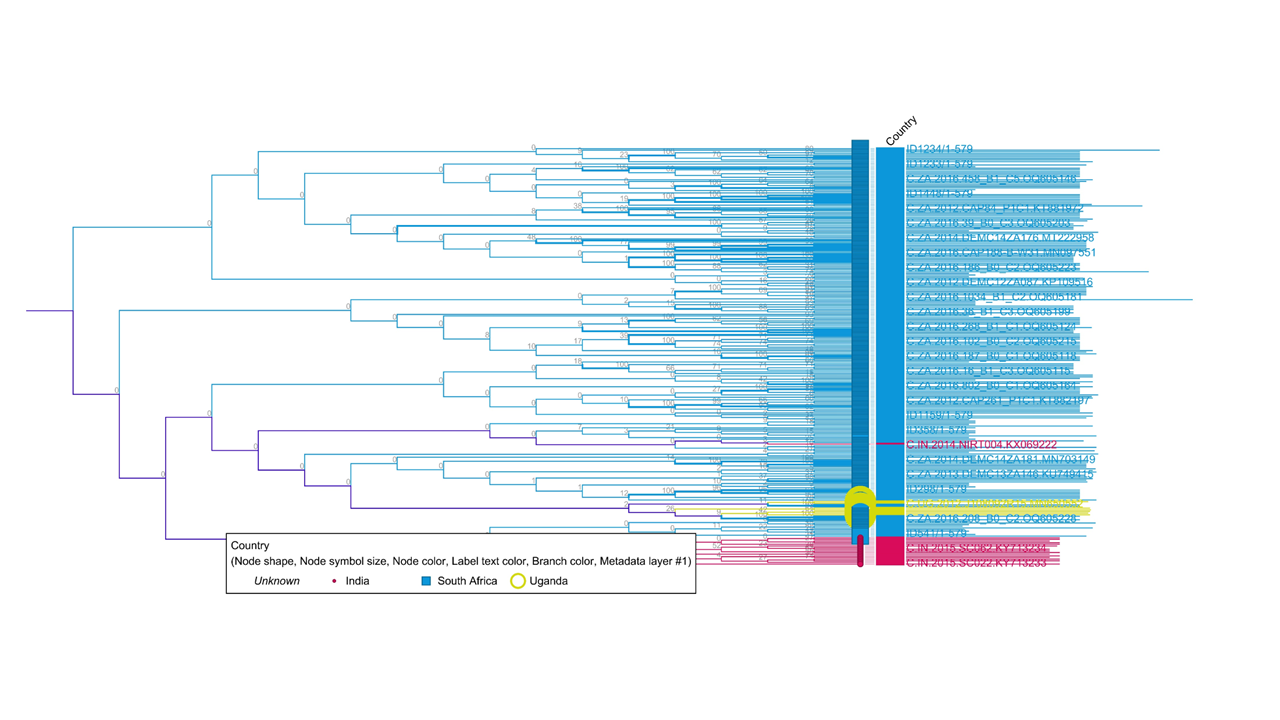

Supplement: Supplementary file 1 — Supplementary file1 (TIF 317 kb) [file 10096_2023_4728_MOESM1_ESM.tif]
