# Supplementary material for: HIV-1 Vif protein sequence variations in South African people living with HIV and their influence on Vif-APOBEC3G interaction
Source: Eur J Clin Microbiol Infect Dis. 2023 Dec 11;43(2):325–38. doi: 10.1007/s10096-023-04728-0 (PMC10821834; doi:10.1007/s10096-023-04728-0)
Supplement: Supplementary file 2 — Supplementary file2 (DOCX 467 kb) [file 10096_2023_4728_MOESM2_ESM.docx]

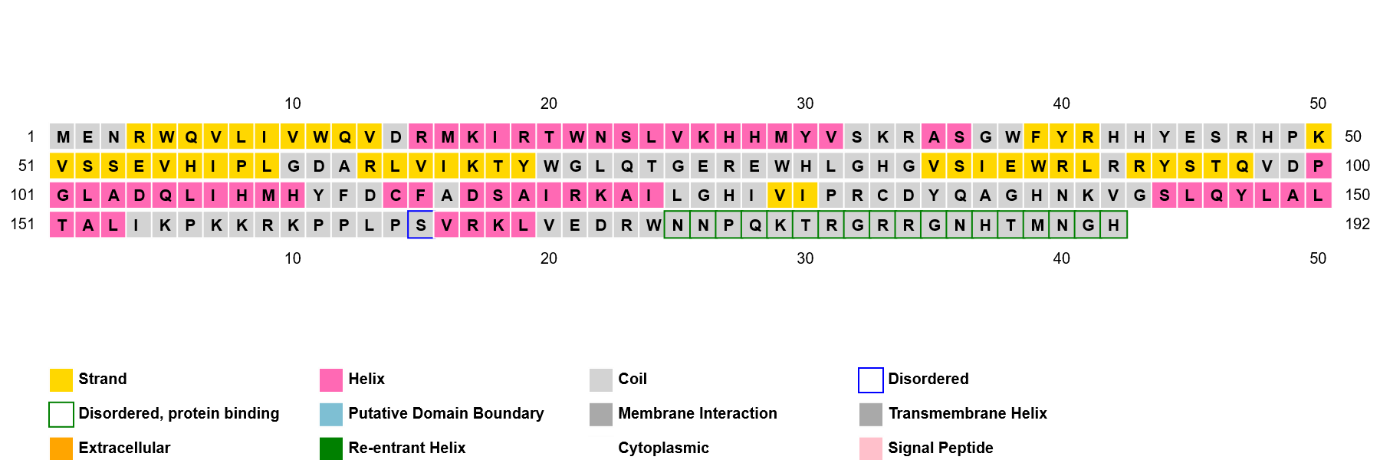


(B)


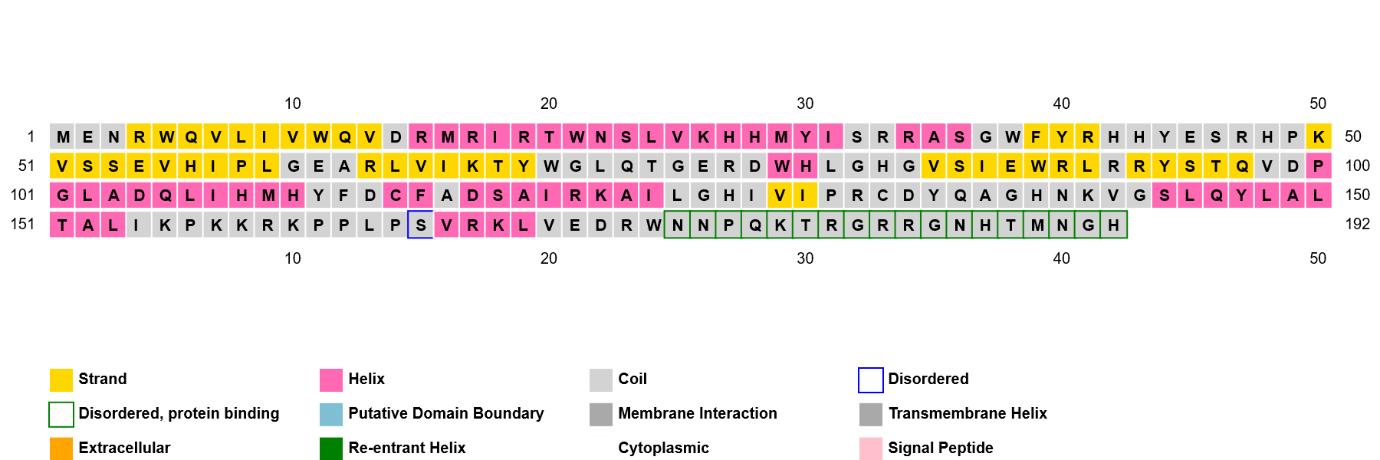


(C)


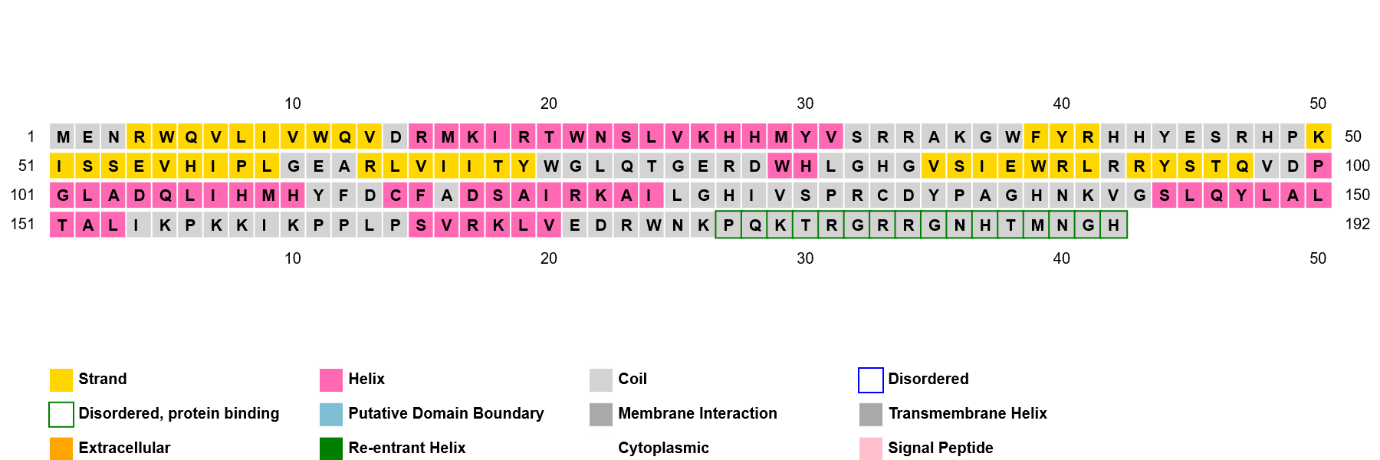


(D)


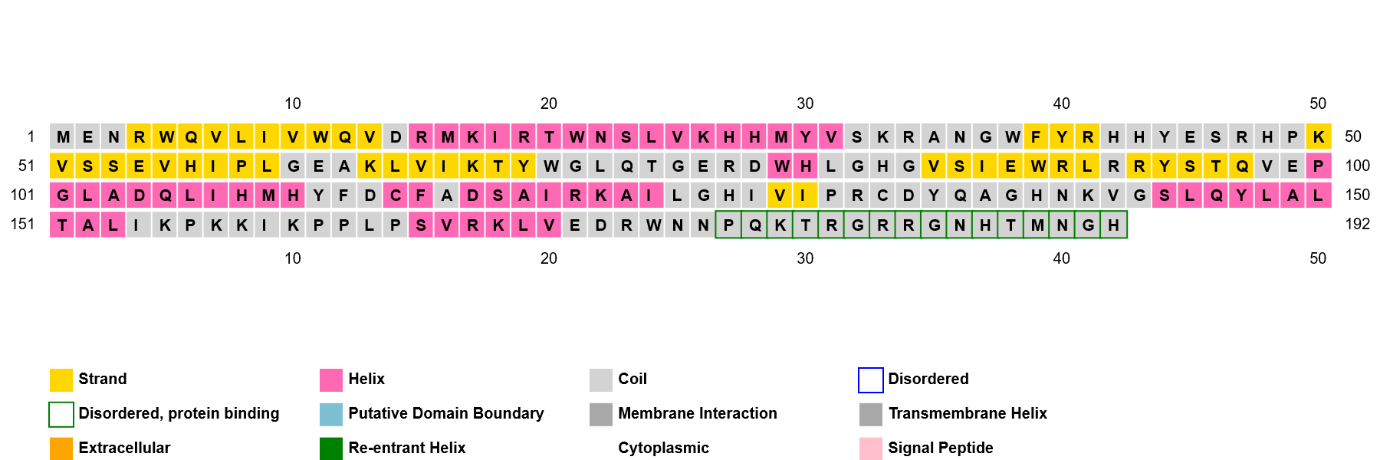


(E)


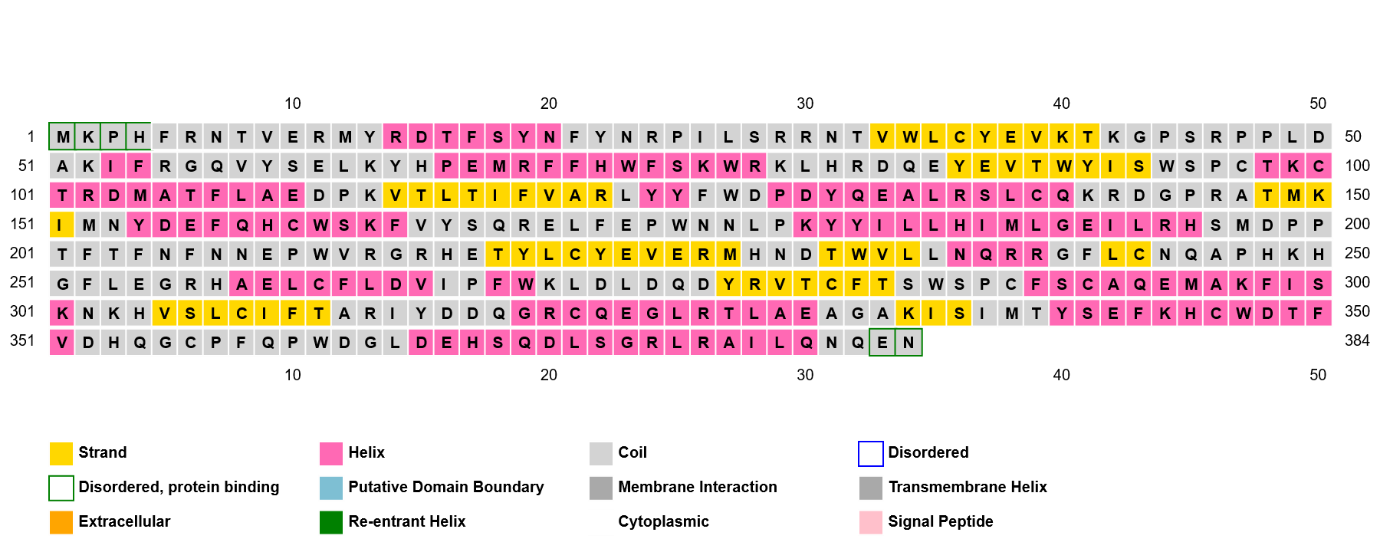


**Supplementary Figure 1A- Figure 1E:** Secondary strcuture prediction for (A) HIV-1C_SA-s, (B) HIV-1C_SA-LA, (C) HIV-1C_UG-LA and (D) HIV-1C_IN-lA and (E) APOBEC3G
