# Supplementary material for: HIV-1 Vif protein sequence variations in South African people living with HIV and their influence on Vif-APOBEC3G interaction
Source: Eur J Clin Microbiol Infect Dis. 2023 Dec 11;43(2):325–38. doi: 10.1007/s10096-023-04728-0 (PMC10821834; doi:10.1007/s10096-023-04728-0)
Supplement: Supplementary file 3 — Supplementary file3 (DOCX 16 kb) [file 10096_2023_4728_MOESM3_ESM.docx]

Supplementary Table 1: Sequences for Vif protein sequences

| Subtype | Sequence |
| --- | --- |
| HIV-1C SA-s | MENRWQVLIVWQVDRMKIRTWNSLVKHHMYVSKRASGWFYRHHYESRHPKVSSEVHIPLGDARLVIKTYWGLQTGEREWHLGHGVSIEWRLRRYSTQVDPGLADQLIHMHYFDCFADSAIRKAILGHIVIPRCDYQAGHNKVGSLQYLALTALIKPKKRKPPLPSVRKLVEDRWNNPQKTRGRRGNHTMNGH |
| HIV-1C SA-LA | MENRWQVLIVWQVDRMRIRTWNSLVKHHMYISRRASGWFYRHHYESRHPKVSSEVHIPLGEARLVIKTYWGLQTGERDWHLGHGVSIEWRLRRYSTQVDPGLADQLIHMHYFDCFADSAIRKAILGHIVIPRCDYQAGHNKVGSLQYLALTALIKPKKRKPPLPSVRKLVEDRWNNPQKTRGRRGNHTMNGH |
| HIV-1C UG-LA | MENRWQVLIVWQVDRMKIRTWNSLVKHHMYVSRRAKGWFYRHHYESRHPKISSEVHIPLGEARLVIITYWGLQTGERDWHLGHGVSIEWRLRRYSTQVDPGLADQLIHMHYFDCFADSAIRKAILGHIVSPRCDYPAGHNKVGSLQYLALTALIKPKKIKPPLPSVRKLVEDRWNKPQKTRGRRGNHTMNGH |
| HIV-1C IND-LA | MENRWQVLIVWQVDRMKIRTWNSLVKHHMYVSKRANGWFYRHHYESRHPKVSSEVHIPLGEAKLVIKTYWGLQTGERDWHLGHGVSIEWRLRRYSTQVEPGLADQLIHMHYFDCFADSAIRKAILGHIVIPRCDYQAGHNKVGSLQYLALTALIKPKKIKPPLPSVRKLVEDRWNNPQKTRGRRGNHTMNGH |
| Q9HC16 · ABC3G_HUMAN | MKPHFRNTVERMYRDTFSYNFYNRPILSRRNTVWLCYEVKTKGPSRPPLDAKIFRGQVYS  ELKYHPEMRFFHWFSKWRKLHRDQEYEVTWYISWSPCTKCTRDMATFLAEDPKVTLTIFV  ARLYYFWDPDYQEALRSLCQKRDGPRATMKIMNYDEFQHCWSKFVYSQRELFEPWNNLPKYYILLHIMLGEILRHSMDPPTFTFNFNNEPWVRGRHETYLCYEVERMHNDTWVLLNQRRGFLCNQAPHKHGFLEGRHAELCFLDVIPFWKLDLDQDYRVTCFTSWSPCFSCAQEMAKFIS  KNKHVSLCIFTARIYDDQGRCQEGLRTLAEAGAKISIMTYSEFKHCWDTFVDHQGCPFQP  WDGLDEHSQDLSGRLRAILQNQEN |
