# Supplementary material for: HIV-1 Vif protein sequence variations in South African people living with HIV and their influence on Vif-APOBEC3G interaction
Source: Eur J Clin Microbiol Infect Dis. 2023 Dec 11;43(2):325–38. doi: 10.1007/s10096-023-04728-0 (PMC10821834; doi:10.1007/s10096-023-04728-0)
Supplement: Supplementary file 4 — Supplementary file4 (DOCX 23 kb) [file 10096_2023_4728_MOESM4_ESM.docx]

**Supplementary Table 2:** Interacting residues between h3AG-HIV-1C IN-LA

| **HIV-1C IN-LA** | | | | | |
| --- | --- | --- | --- | --- | --- |
| **H3AG amino acid** | **Position** | **Chain** | **Vif amino acid** | **Position** | **Chain** |
| ASP | 198 | A | ASP | 14 | B |
| PRO | 200 | A | ASP | 14 | B |
| THR | 201 | A | ASP | 14 | B |
| HIS | 195 | A | ARG | 15 | B |
| ASP | 198 | A | ARG | 15 | B |
| PRO | 199 | A | ARG | 15 | B |
| PRO | 200 | A | ARG | 15 | B |
| PRO | 129 | A | MET | 16 | B |
| HIS | 195 | A | MET | 16 | B |
| ASP | 198 | A | MET | 16 | B |
| THR | 201 | A | MET | 16 | B |
| TYR | 219 | A | MET | 16 | B |
| TRP | 127 | A | ILE | 18 | B |
| PHE | 126 | A | ARG | 19 | B |
| TRP | 127 | A | ARG | 19 | B |
| ASP | 128 | A | ARG | 19 | B |
| PRO | 129 | A | ARG | 19 | B |
| GLN | 132 | A | ARG | 19 | B |
| MET | 188 | A | ARG | 19 | B |
| GLU | 191 | A | ARG | 19 | B |
| ILE | 192 | A | ARG | 19 | B |
| HIS | 195 | A | ARG | 19 | B |
| TRP | 127 | A | THR | 20 | B |
| ASP | 128 | A | THR | 20 | B |
| PRO | 129 | A | THR | 20 | B |
| TYR | 125 | A | ASN | 22 | B |
| TRP | 127 | A | ASN | 22 | B |
| TYR | 125 | A | SER | 23 | B |
| TRP | 127 | A | SER | 23 | B |
| ASP | 128 | A | SER | 23 | B |
| PRO | 129 | A | SER | 23 | B |
| TYR | 125 | A | LEU | 24 | B |
| ASP | 128 | A | LEU | 24 | B |
| ILE | 26 | A | LYS | 26 | B |
| ARG | 122 | A | LYS | 26 | B |
| TYR | 124 | A | LYS | 26 | B |
| TYR | 125 | A | LYS | 26 | B |
| PHE | 126 | A | LYS | 26 | B |
| TRP | 127 | A | LYS | 26 | B |
| TYR | 125 | A | HIS | 27 | B |
| HIS | 250 | A | HIS | 27 | B |
| ILE | 26 | A | TYR | 30 | B |
| SER | 28 | A | TYR | 30 | B |
| TYR | 124 | A | TYR | 30 | B |
| TYR | 59 | A | VAL | 31 | B |
| TYR | 124 | A | VAL | 31 | B |
| TYR | 125 | A | VAL | 31 | B |
| ILE | 26 | A | TYR | 40 | B |
| PHE | 126 | A | TYR | 40 | B |
| TRP | 127 | A | TYR | 40 | B |
| ILE | 26 | A | ARG | 41 | B |
| ARG | 24 | A | HIS | 42 | B |
| PRO | 25 | A | HIS | 42 | B |
| ILE | 26 | A | HIS | 42 | B |
| LEU | 184 | A | HIS | 42 | B |
| PRO | 25 | A | HIS | 43 | B |
| ILE | 26 | A | HIS | 43 | B |
| PHE | 126 | A | HIS | 43 | B |
| TRP | 127 | A | HIS | 43 | B |
| LEU | 184 | A | HIS | 43 | B |
| MET | 188 | A | HIS | 43 | B |
| LYS | 180 | A | TYR | 44 | B |
| LEU | 184 | A | TYR | 44 | B |
| ILE | 187 | A | TYR | 44 | B |
| LYS | 180 | A | GLU | 45 | B |
| TRP | 127 | A | SER | 52 | B |
| TRP | 127 | A | SER | 53 | B |
| ASP | 198 | A | GLU | 76 | B |
| PRO | 199 | A | GLU | 76 | B |
| PRO | 200 | A | GLU | 76 | B |
| GLN | 354 | A | ARG | 77 | B |
| PRO | 199 | A | ASP | 78 | B |
| ASP | 352 | A | ASP | 78 | B |
| GLN | 354 | A | ASP | 78 | B |
| GLU | 191 | A | TRP | 79 | B |
| ARG | 194 | A | TRP | 79 | B |
| HIS | 195 | A | TRP | 79 | B |
| ASP | 352 | A | TRP | 79 | B |
| GLN | 354 | A | TRP | 79 | B |
| GLU | 191 | A | HIS | 80 | B |
| GLN | 354 | A | HIS | 80 | B |
| TRP | 127 | A | HIS | 83 | B |
| LYS | 249 | A | LYS | 158 | B |
| LYS | 249 | A | ILE | 159 | B |
| HIS | 248 | A | LYS | 160 | B |
| LYS | 249 | A | LYS | 160 | B |
| HIS | 250 | A | LYS | 160 | B |
| HIS | 248 | A | PRO | 161 | B |
| LYS | 249 | A | PRO | 161 | B |
| HIS | 250 | A | PRO | 161 | B |
| HIS | 248 | A | PRO | 162 | B |
| PRO | 200 | A | TRP | 174 | B |
| THR | 201 | A | TRP | 174 | B |
| PHE | 204 | A | TRP | 174 | B |
| ARG | 213 | A | TRP | 174 | B |
| GLY | 214 | A | TRP | 174 | B |
| ARG | 213 | A | ASN | 175 | B |
| GLY | 214 | A | ASN | 175 | B |
