# Supplementary material for: HIV-1 Vif protein sequence variations in South African people living with HIV and their influence on Vif-APOBEC3G interaction
Source: Eur J Clin Microbiol Infect Dis. 2023 Dec 11;43(2):325–38. doi: 10.1007/s10096-023-04728-0 (PMC10821834; doi:10.1007/s10096-023-04728-0)
Supplement: Supplementary file 5 — Supplementary file5 (DOCX 23 kb) [file 10096_2023_4728_MOESM5_ESM.docx]

**Supplementary Table 3:** Interacting residues between h3AG-HIV-1C SA-LA

| **HIV-1C IN-LA** | | | | | |
| --- | --- | --- | --- | --- | --- |
| **H3AG amino acid** | **Position** | **Chain** | **Vif amino acid** | **Position** | **Chain** |
| TRP | 127 | A | ILE | 18 | B |
| TRP | 127 | A | ARG | 19 | B |
| LEU | 184 | A | ARG | 19 | B |
| ILE | 26 | A | ARG | 19 | B |
| TRP | 127 | A | ASN | 22 | B |
| LEU | 184 | A | SER | 23 | B |
| ILE | 187 | A | SER | 23 | B |
| MET | 188 | A | LYS | 26 | B |
| GLN | 132 | A | LYS | 26 | B |
| PHE | 126 | A | LYS | 26 | B |
| ILE | 187 | A | LYS | 26 | B |
| ILE | 192 | A | LYS | 26 | B |
| GLU | 191 | A | LYS | 26 | B |
| ILE | 187 | A | HIS | 27 | B |
| GLN | 354 | A | MET | 29 | B |
| GLU | 191 | A | MET | 29 | B |
| HIS | 195 | A | MET | 29 | B |
| GLN | 354 | A | TYR | 30 | B |
| GLY | 190 | A | TYR | 30 | B |
| GLU | 191 | A | TYR | 30 | B |
| HIS | 195 | A | TYR | 30 | B |
| ARG | 194 | A | TYR | 30 | B |
| SER | 196 | A | TYR | 30 | B |
| ASP | 352 | A | TYR | 30 | B |
| ILE | 192 | A | TYR | 30 | B |
| ILE | 187 | A | ILE | 31 | B |
| GLY | 190 | A | ILE | 31 | B |
| GLU | 191 | A | ILE | 31 | B |
| HIS | 186 | A | ILE | 31 | B |
| GLN | 354 | A | ALA | 35 | B |
| GLN | 354 | A | SER | 36 | B |
| GLN | 354 | A | GLY | 37 | B |
| GLN | 354 | A | TRP | 38 | B |
| ASP | 198 | A | PHE | 39 | B |
| PRO | 199 | A | PHE | 39 | B |
| HIS | 353 | A | PHE | 39 | B |
| ASP | 352 | A | PHE | 39 | B |
| PRO | 200 | A | PHE | 39 | B |
| GLN | 354 | A | PHE | 39 | B |
| HIS | 195 | A | TYR | 40 | B |
| TRP | 127 | A | TYR | 40 | B |
| GLU | 191 | A | TYR | 40 | B |
| ASP | 198 | A | TYR | 40 | B |
| ASP | 198 | A | ARG | 41 | B |
| PRO | 200 | A | ARG | 41 | B |
| THR | 201 | A | ARG | 41 | B |
| GLN | 132 | A | HIS | 42 | B |
| PHE | 126 | A | HIS | 42 | B |
| ASP | 128 | A | HIS | 42 | B |
| PRO | 129 | A | HIS | 42 | B |
| ILE | 192 | A | HIS | 42 | B |
| GLU | 191 | A | HIS | 42 | B |
| HIS | 195 | A | HIS | 42 | B |
| TRP | 127 | A | HIS | 42 | B |
| ASP | 128 | A | HIS | 43 | B |
| PRO | 129 | A | HIS | 43 | B |
| HIS | 248 | A | HIS | 43 | B |
| TRP | 127 | A | HIS | 43 | B |
| GLN | 245 | A | TYR | 44 | B |
| GLU | 217 | A | TYR | 44 | B |
| HIS | 248 | A | TYR | 44 | B |
| PRO | 129 | A | TYR | 44 | B |
| PRO | 247 | A | TYR | 44 | B |
| TYR | 219 | A | TYR | 44 | B |
| ALA | 246 | A | TYR | 44 | B |
| ASP | 128 | A | TYR | 44 | B |
| ASN | 244 | A | TYR | 44 | B |
| TYR | 219 | A | GLU | 45 | B |
| PRO | 129 | A | GLU | 45 | B |
| ASP | 198 | A | GLU | 45 | B |
| THR | 201 | A | GLU | 45 | B |
| GLU | 217 | A | ARG | 47 | B |
| ARG | 215 | A | ARG | 47 | B |
| HIS | 216 | A | ARG | 47 | B |
| ARG | 213 | A | ARG | 47 | B |
| GLY | 214 | A | ARG | 47 | B |
| PHE | 204 | A | ARG | 47 | B |
| TRP | 127 | A | SER | 52 | B |
| TRP | 127 | A | SER | 53 | B |
| PRO | 200 | A | HIS | 56 | B |
| ASP | 198 | A | HIS | 56 | B |
| PRO | 200 | A | ARG | 63 | B |
| TRP | 127 | A | THR | 68 | B |
| TRP | 127 | A | TRP | 70 | B |
| SER | 28 | A | TRP | 79 | B |
| ILE | 26 | A | HIS | 80 | B |
| GLN | 359 | A | ARG | 92 | B |
